# The Role of Systemic Health Indicators, Including C-Reactive Protein and eGFR, in Predicting Periodontal Disease: A Longitudinal Study

**DOI:** 10.3390/ijms26020741

**Published:** 2025-01-16

**Authors:** Amr Sayed Ghanem

**Affiliations:** Department of Health Informatics, Faculty of Health Sciences, University of Debrecen, 4032 Debrecen, Hungary; aghanem@etk.unideb.hu

**Keywords:** c-reactive protein, estimated glomerular filtration rate, CRP, eGFR, periodontitis, periodontal disease, atherosclerosis, oral disease, dyslipidemia

## Abstract

C-reactive protein (CRP) and estimated glomerular filtration rate (eGFR) are key biomarkers reflecting systemic inflammation and metabolic dysfunction. This study explored systemic and oral health indicators, including CRP and eGFR, as potential factors associated with periodontitis, using a longitudinal clinical dataset comprising 23,742 records from patients identified by ICD-10 codes between 2015 and 2022. Univariate Cox analysis and Gompertz models, selected based on AIC and BIC after evaluating alternative models, were employed to assess the predictive roles of CRP and eGFR in periodontitis incidence, adjusting for oral and systemic health factors. Elevated CRP (>15 mg/L) and reduced eGFR (<60 mL/min/1.73 m^2^) were significant predictors of periodontitis, with hazard ratios (HR) of 1.36 [1.05–1.77] and 1.39 [1.08–1.78], respectively. Atherosclerosis (HR: 2.12 [1.11–4.06]), diseases of the hard tissues of the teeth (HR: 7.30 [5.45–9.78]), and disorders of the teeth and supporting structures (HR: 3.02 [2.05–4.43]) also demonstrated strong predictive associations. CRP and eGFR emerged as potential biomarkers for predicting periodontitis, enabling early interventions to prevent tooth loss and systemic complications. Patients with chronic kidney disease, atherosclerotic heart disease, and lipid metabolism disorders are at higher risk, emphasizing the need for integrated care addressing both systemic and oral health factors.

## 1. Introduction

Oral diseases represent the most widespread health conditions globally, affecting approximately 3.5 billion people in 2019, leading to a global prevalence of approximately 45%, with consistent patterns observed across World Bank income classifications and WHO regions [1]. Among these, untreated caries in permanent teeth ranks highest, with around 2 billion cases, followed by severe periodontal disease, affecting nearly 1 billion individuals [2]. Other prevalent conditions include untreated caries in deciduous teeth (510 million cases) and edentulism (350 million cases). Notably, the global burden of oral diseases surpasses the combined case numbers of five major non-communicable diseases (NCDs), including cardiovascular diseases, diabetes, and cancer. Between 1990 and 2019, oral disease case numbers increased by 50%, surpassing the 45% growth in the global population during the same period [3].

Oral diseases affect individuals across the lifespan, with specific conditions peaking in different age groups. Caries in deciduous teeth is most common around age six, while caries in permanent teeth peaks in adolescence and early adulthood. Severe periodontal disease reaches its highest prevalence around the age of 60, reflecting age-dependent patterns of clinical attachment loss (CAL) observed across diverse populations, despite differences in overall severity. Empirical evidence suggests that CAL thresholds indicating disproportionate severity of periodontitis by age are feasible and applicable across populations, while newer generations of older adults exhibit a higher prevalence and severity of chronic periodontitis, further influenced by factors such as male sex, low household income, lack of dental insurance, and smoking [4,5]. The chronic, progressive, and cumulative nature of oral diseases often results in significant physical, social, and psychological consequences, including pain, reduced productivity, and impaired self-esteem [6,7].

Oral health and systemic health share complex bidirectional relationships mediated by common biological and behavioral risk factors, including inflammation, microbiome dysregulation, and systemic biomarkers like C-reactive protein (CRP) and estimated glomerular filtration rate (eGFR). Evidence consistently links severe periodontal disease with elevated CRP levels, which contribute to the progress of other non-communicable diseases [8,9,10,11,12]. CRP, an acute-phase reactant synthesized by hepatocytes in response to pro-inflammatory cytokines such as interleukin-6 and tumor necrosis factor-alpha, is a key biomarker of systemic inflammation associated with periodontal disease [13,14]. Periodontitis, initiated by dysbiotic bacterial biofilms, induces local and systemic cytokine release, leading to elevated CRP levels that correlate with disease severity, including clinical attachment loss and bleeding on probing [15], with scientific literature pointing towards its significant association in specific subpopulations, such as individuals with obesity [16]. Chronically elevated CRP is not merely a marker but can contribute to the pathogenesis of systemic diseases such as atherosclerosis, cardiovascular disease, diabetes, and chronic kidney disease (CKD) by promoting endothelial dysfunction, oxidative stress, and immune dysregulation [17]. CKD exacerbates periodontitis through systemic inflammation, immune dysregulation, and impaired oral microbiome balance. Declining eGFR, a marker of CKD severity, correlates with increased pro-inflammatory cytokines such as IL-6 and TNF-α, promoting gingival inflammation, periodontal pocket formation, and alveolar bone resorption [18]. CKD-related immune suppression further predisposes to dysbiosis and periodontal tissue destruction. Studies consistently demonstrate a positive association between CKD severity and periodontitis, with evidence supporting the efficacy of periodontal therapy in improving systemic inflammation and eGFR [19,20].

This study aimed to address gaps in literature by utilizing a robust time-to-event analysis model to evaluate the time-dependent associations between systemic health indicators, including inflammatory biomarkers (CRP), renal function biomarkers (eGFR), metabolic and cardiovascular conditions, and specific dental pathologies with periodontitis risk. Unlike prior research, it explores a range of systemic and dental factors, highlighting the role of systemic multimorbidity and offering a broader understanding of oral-systemic health interactions.

## 2. Results

At baseline, depicted in Table 1, the study sample consisted of 4833 participants with a mean age of 50.43 years (SD: 15.40), a median of 51 years [IQR: 40–62], and an age range of 0 to 89 years. Females constituted 54.60% (*n* = 2637) of the sample, while males accounted for 45.40% (*n* = 2193). CRP levels were within the normal range (≤15 mg/L) in 74.31% (*n* = 295) of cases, while 25.69% (*n* = 102) had elevated CRP levels (>15 mg/L). Estimated glomerular filtration rate (eGFR) was ≥60 mL/min/1.73 m^2^ in 80.25% (*n* = 317) of participants, with 19.75% (*n* = 78) showing eGFR < 60 mL/min/1.73 m^2^, indicative of kidney disease. GOT levels were normal (<40 IU) in 91.83% (*n* = 528) and elevated (≥40 IU) in 8.17% (*n* = 47). GPT levels were normal (<40 IU) in 87.67% (*n* = 576) and elevated (≥40 IU) in 12.33% (*n* = 81).

Among systemic and oral health variables, 97.33% (*n* = 4704) were absent for disorders of lipoprotein metabolism (E78), while 2.67% (*n* = 129) were present. Atherosclerosis (I70) was observed in 0.77% (*n* = 37), with 99.23% (*n* = 4796) being absent. Disorders of tooth development and eruption (K00) were present in 1.37% (*n* = 66), while 98.63% (*n* = 4767) were absent. Other diseases of hard tissues of teeth (K03) were present in 15.93% (*n* = 770) and absent in 84.07% (*n* = 4063). Diseases of pulp and periapical tissues (K04) were present in 3.04% (*n* = 147), and 96.96% (*n* = 4686) were absent. Other disorders of gingiva and edentulous alveolar ridge (K06) were present in 1.59% (*n* = 77), with 98.41% (*n* = 4756) absent. Other specified disorders of teeth and supporting structures (K08) were present in 3.46% (*n* = 167) and absent in 96.54% (*n* = 4666). Type 2 diabetes mellitus (E11) was diagnosed in 1.86% (*n* = 90), while 98.14% (*n* = 4743) did not have the condition. Essential hypertension (I10) was present in 6.95% (*n* = 336) of participants, with 93.05% (*n* = 4497) being free of the condition. Out of the total 4833 patients included in the study, gender information was available for 4830 patients. Measurements for CRP were available for 397 patients, eGFR for 395 patients, GOT for 575 patients, and GPT for 657 patients.

**Figure 1:**
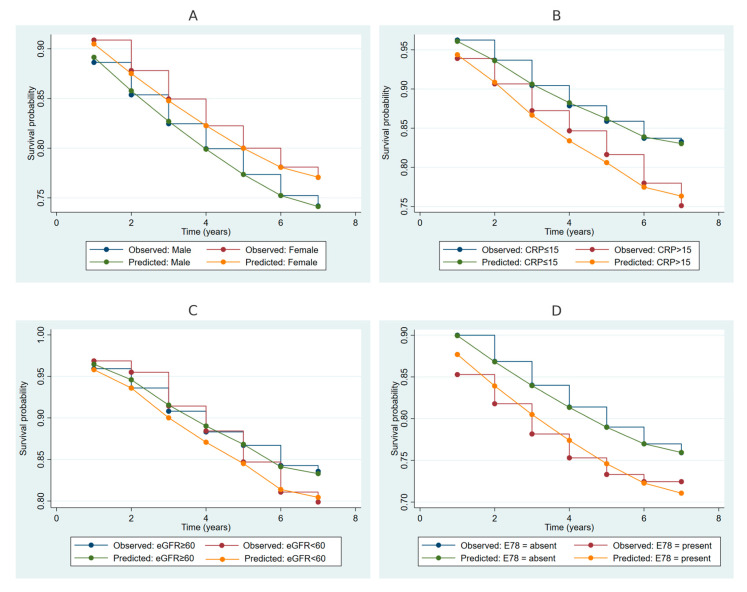
Kaplan–Meier survival curves for periodontitis risk stratified by gender, CRP levels, eGFR categories, and disorders of lipoprotein metabolism. Note: Kaplan–Meier survival curves were generated using observed and predicted survival probabilities for periodontitis across subgroups. Panel (**A**): Stratification by gender (male and female). Panel (**B**): Stratification by C-reactive protein (CRP ≤ 15 mg/L vs. >15 mg/L). Panel (**C**): Stratification by estimated glomerular filtration rate (eGFR ≥ 60 mL/min/1.73 m^2^ vs. <60 mL/min/1.73 m^2^). Panel (**D**): Stratification by the presence or absence of disorders of lipoprotein metabolism (E78, ICD-10 code). CRP, C-reactive protein; eGFR, estimated glomerular filtration rate.

Figure 1 illustrates Kaplan–Meier survival curves for the risk of periodontitis over the follow-up period, stratified by significant variables. Figure 1A depicts survival probabilities by gender, with males and females showing similar trends in observed and predicted survival, although a slight divergence is noted in later years. Figure 1B highlights the stratification by CRP levels, where individuals with elevated CRP (>15 mg/L) exhibit lower survival probabilities compared to those with normal CRP (≤15 mg/L). Figure 1C compares survival based on estimated glomerular filtration rate (eGFR), showing individuals with eGFR < 60 mL/min/1.73 m^2^ experiencing reduced survival probabilities relative to those with normal eGFR (≥60 mL/min/1.73 m^2^). Figure 1D illustrates the survival curves stratified by the presence of disorders of lipoprotein metabolism (E78), with individuals presenting with E78 demonstrating notably lower survival probabilities compared to those without the condition.

**Figure 2:**
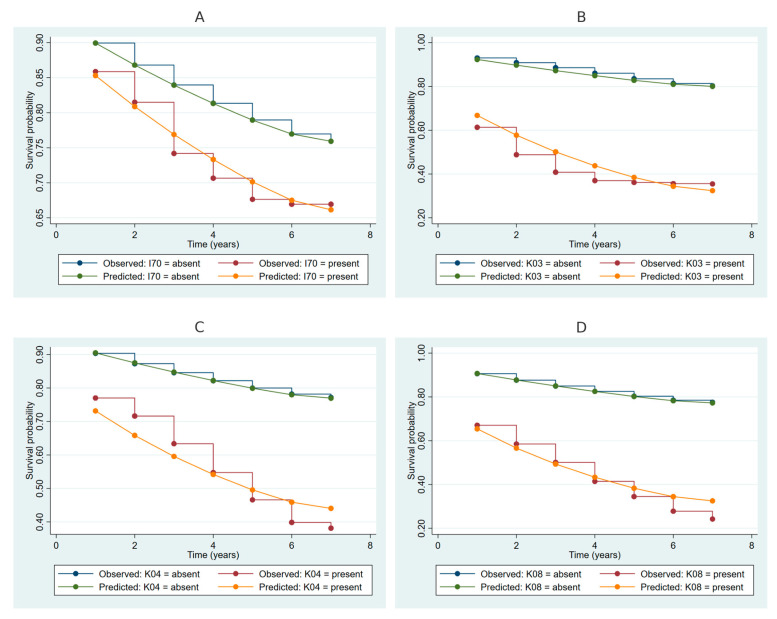
Kaplan–Meier survival curves for periodontitis risk stratified by ICD-10 codes I70, K03, K04, and K08. Note: Panel (**A**): Stratification by the presence or absence of atherosclerosis (I70). Panel (**B**): Stratification by other diseases of hard tissues of teeth (K03). Panel (**C**): Stratification by diseases of pulp and periapical tissues (K04). Panel (**D**): Stratification by other specified disorders of teeth and supporting structures (K08).

In Figure 2, Figure 2A illustrates that participants with atherosclerosis (I70) demonstrated lower survival probabilities compared to those without the condition, with divergence in observed and predicted curves becoming more pronounced over time. Figure 2B shows that participants with other diseases of hard tissues of teeth (K03) exhibited substantially lower survival probabilities than those without these conditions, with predicted survival closely mirroring observed survival. Figure 2C highlights that participants with diseases of pulp and periapical tissues (K04) had significantly reduced survival probabilities compared to those without these diseases. Finally, Figure 2D reveals that participants with other specified disorders of the teeth and supporting structures (K08) experienced the steepest decline in survival probabilities, with observed and predicted curves aligning consistently over the follow-up period.

Table 2 summarizes the univariate Cox regression analysis results for periodontitis. Females showed a lower relative hazard (RH = 0.94) compared to males (RH = 1.09, *p* < 0.001). Participants with high CRP levels (>15 mg/L) demonstrated an increased relative hazard (RH = 1.34) compared to those with normal levels (≤15 mg/L, RH = 0.92, *p* < 0.001). While participants with kidney disease (eGFR < 60 mL/min/1.73 m^2^) had a higher relative hazard (RH = 1.12) compared to those with normal eGFR (≥60 mL/min/1.73 m^2^, RH = 0.94), this result was not statistically significant (*p* = 0.072). Elevated GPT levels (>40 IU) were associated with a higher relative hazard (RH = 1.25, *p* = 0.024) compared to normal levels. Disorders of lipoprotein metabolism (E78) were linked to a higher relative hazard (RH = 1.24, *p* = 0.022). Atherosclerosis (I70) significantly increased the relative hazard (RH = 1.5, *p* = 0.004). Disorders of tooth development and eruption (K00) were associated with a marked increase in relative hazard (RH = 2.79, *p* < 0.001), as were other diseases of hard tissues of teeth (K03, RH = 4.5, *p* < 0.001), diseases of pulp and periapical tissues (K04, RH = 3.03, *p* < 0.001), other disorders of gingiva and edentulous alveolar ridge (K06, RH = 4.3, *p* < 0.001), and other specified disorders of teeth and supporting structures (K08, RH = 4.19, *p* < 0.001). Type 2 diabetes mellitus (E11) was also associated with an increased relative hazard (RH = 1.4, *p* = 0.002), and essential hypertension (I10) significantly elevated the relative hazard (RH = 1.45, *p* < 0.001).

**Figure 3:**
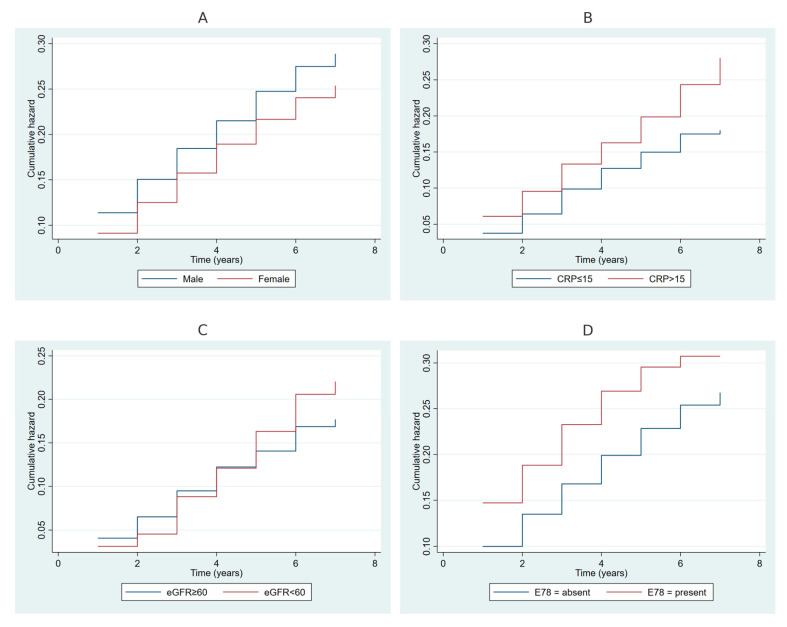
(**A**) Cumulative hazard of periodontitis over time, stratified by gender (blue line: Male; red line: Female). (**B**) Cumulative hazard of periodontitis over time, stratified by C-reactive protein (CRP) level (blue line: CRP ≤ 15 mg/L; red line: CRP > 15 mg/L). (**C**) Cumulative hazard of periodontitis over time, stratified by estimated glomerular filtration rate (eGFR) (blue line: eGFR ≥ 60 mL/min/1.73 m²; red line: eGFR < 60 mL/min/1.73 m²). (**D**) Cumulative hazard of periodontitis over time, stratified by E78 status (blue line: absent; red line: present). Note: The cumulative hazard curves were generated using the Nelson–Aalen estimator for periodontitis, and E78 refers to ICD-10 codes for disorders of lipoprotein metabolism.

Figure 3 presents cumulative hazard plots for periodontitis stratified by selected variables. Figure 3A displays the cumulative hazard for periodontitis by gender, showing higher cumulative hazards among males compared to females over the follow-up period. Figure 3B compares cumulative hazards by CRP levels, where participants with high CRP levels (>15 mg/L) exhibited consistently higher cumulative hazards than those with normal levels (≤15 mg/L). Figure 3C illustrates the stratification by estimated glomerular filtration rate (eGFR), with individuals having kidney disease (eGFR < 60 mL/min/1.73 m^2^) showing increased cumulative hazards compared to those with normal eGFR (≥60 mL/min/1.73 m^2^). Finally, Figure 3D depicts cumulative hazards by the presence of disorders of lipoprotein metabolism (E78), highlighting higher hazards in participants with this condition compared to those without.

**Figure 4:**
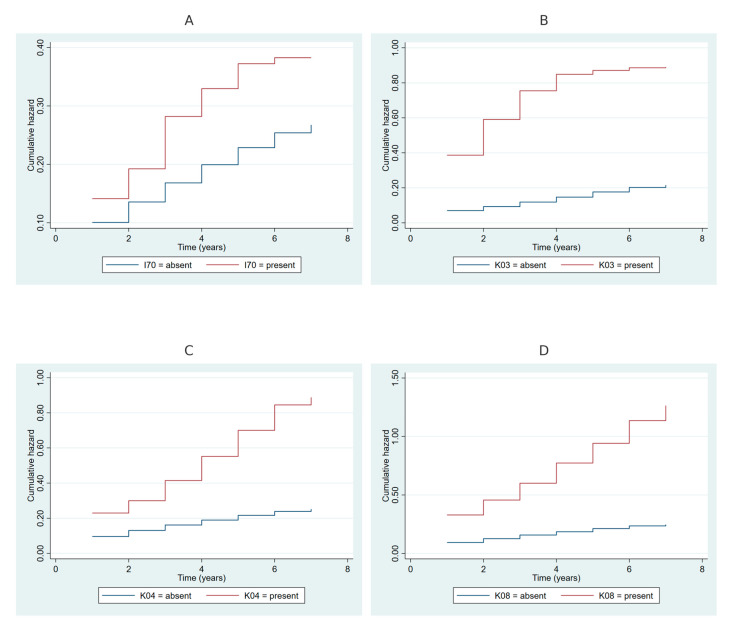
(**A**) Cumulative hazard of periodontitis, stratified by atherosclerosis (I70) status (blue: absent; red: present). (**B**) Stratified by other diseases of hard tissues of teeth (K03). (**C**) Stratified by diseases of pulp and periapical tissues (K04). (**D**) Stratified by other specified disorders of teeth and supporting structures (K08). Cumulative hazard curves were generated using the Nelson–Aalen estimator. I70, K03, K04, and K08 refer to the corresponding ICD-10 codes.

Figure 4 illustrates cumulative hazard plots for periodontitis stratified by oral and systemic health conditions. Figure 4A compares participants with and without atherosclerosis (I70), showing consistently higher cumulative hazards for those with atherosclerosis over the follow-up period. Figure 4B depicts cumulative hazards stratified by the presence of other diseases of hard tissues of teeth (K03), with significantly elevated hazards observed among participants with this condition compared to those without. Figure 4C presents cumulative hazards for diseases of pulp and periapical tissues (K04), revealing increased risks for participants with these conditions. Lastly, Figure 4D demonstrates cumulative hazards stratified by the presence of other specified disorders of teeth and supporting structures (K08), with a marked increase in cumulative hazard among affected individuals.

The multivariable Gompertz model, as shown in Table 3, revealed that age was associated with a slight reduction in the hazard of periodontitis (HR: 0.99 [95% CI: 0.98–0.99], *p* = 0.001). Gender showed a marginal association, with females exhibiting a lower hazard compared to males (HR: 0.79 [95% CI: 0.63–1.00], *p* = 0.054). Elevated C-reactive protein levels (>15 mg/L) significantly increased the hazard (HR: 1.36 [95% CI: 1.05–1.77], *p* = 0.02), as did reduced eGFR (<60 mL/min/1.73 m^2^) (HR: 1.39 [95% CI: 1.08–1.78], *p* = 0.01). No significant associations were observed for elevated GOT (HR: 0.89 [95% CI: 0.57–1.38], *p* = 0.604) or GPT (HR: 1.04 [95% CI: 0.72–1.48], *p* = 0.85). Atherosclerosis (I70) was associated with a higher hazard (HR: 2.12 [95% CI: 1.11–4.06], *p* = 0.023), while disorders of lipoprotein metabolism (E78) were not significantly associated (HR: 0.75 [95% CI: 0.40–1.40], *p* = 0.361). Among oral health variables, other diseases of hard tissues of teeth (K03) presented the highest hazard (HR: 7.30 [95% CI: 5.45–9.78], *p* < 0.001), followed by other specified disorders of teeth and supporting structures (K08) (HR: 3.02 [95% CI: 2.05–4.43], *p* < 0.001) and diseases of pulp and periapical tissues (K04) (HR: 1.91 [95% CI: 1.14–3.20], *p* = 0.014). Disorders of tooth development and eruption (K00), other disorders of gingiva and edentulous alveolar ridge (K06), type 2 diabetes mellitus (E11), and essential hypertension (I10) showed no significant associations (*p*-values of 0.729, 0.357, 0.673, and 0.755, respectively).

## 3. Discussion

This study is the first in assessing the combined predictive capacity of CRP eGFR in periodontal disease accounting for both oral and systemic diseases. The study’s findings indicated the emergence of elevated CRP and low eGFR as predictor biomarkers for periodontitis. Atherosclerosis and lipoprotein metabolism disorders were both risk factors for periodontitis, in addition to diseases of the hard tissues of teeth, pulp and periapical tissues, and supporting structures, which all demonstrated strong predictive value for periodontal disease onset.

Jasuma Rai et al. (2023) identified CRP as an indicator of periodontal disease severity, reporting a positive correlation between serum CRP levels and the periodontitis severity grading scale [21]. Their findings emphasized the association between systemic inflammatory markers and periodontal inflammation. The present study builds upon these findings, demonstrating that elevated CRP levels are significantly associated with an increased risk of periodontitis. Unlike the aforementioned cross-sectional study, this analysis employed a longitudinal time-to-event design, leveraging repeated laboratory measurements collected over a seven-year follow-up period. This approach, within the context of a comprehensive systemic and oral health framework, provides a deeper understanding of the temporal dynamics of periodontitis progression. This points to the potential of CRP to serve as a predictor of periodontitis due to its role as an acute-phase reactant that responds to bacterial components involved in periodontal infection [22], such as Porphyromonas gingivalis and Treponema denticola. These pathogens trigger a systemic inflammatory response [23], with CRP acting as an early biomarker of this reaction, reflecting the host’s immune response to microbial invasion and tissue destruction.

Although CRP is a well-established and widely available marker for systemic inflammation, its lack of disease specificity and susceptibility to influence by non-oral inflammatory conditions represent important limitations. As such, its utility as a predictive biomarker for periodontitis must be evaluated in conjunction with other biomarkers and clinical conditions. The estimated glomerular filtration rate, a measure of renal function, is one such biomarker with particular interest. The relationship between kidney function, indicated by eGFR, and periodontitis has been a subject of conflicting evidence. A large follow-up study using data from the Study of Health in Pomerania found no consistent association between periodontitis and decreased kidney function in a cohort of younger adults aged 20 to 59 years [24]. In contrast, a longitudinal study of older Japanese adults reported that CKD significantly increased the risk of periodontal disease progression [25], with an adjusted odds ratio of 1.73 (95% CI: 1.15–2.60). While previous studies did not incorporate CRP or other systemic and oral health-related factors into their analyses, the current study demonstrated that reduced eGFR, in conjunction with elevated CRP, serves as a significant predictor of periodontal disease within a robust methodological framework. Patients with CKD could be considered an at-risk population for periodontitis, as CKD triggers systemic inflammation and immune dysregulation [26], which are key drivers of periodontal disease. Reduced eGFR, a hallmark of CKD, is associated with elevated levels of pro-inflammatory cytokines such as interleukin-6 and tumor necrosis factor-alpha, which stimulate CRP production [27]. Elevated CRP reflects heightened systemic inflammation that exacerbates periodontal tissue destruction by promoting bacterial colonization, impairing immune responses, and delaying tissue repair. Furthermore, CKD-related metabolic disturbances, including oxidative stress and uremic toxin accumulation, weaken periodontal defenses, increasing susceptibility to infection and disease progression [28].

Building on the systemic nature of periodontitis, the current study’s findings also highlighted the interaction between inflammation, vascular health, and lipid metabolism in predicting disease onset. Beyond CRP and eGFR, disorders of lipid metabolism and atherosclerosis emerged as significant predictors of periodontal disease. Most existing research highlights periodontitis as a contributing factor to systemic conditions, including atherosclerosis and disorders of lipid metabolism, largely through its role in propagating systemic inflammation [29,30,31]. However, the current study offers a different perspective, demonstrating that these systemic conditions themselves are significant predictors of periodontitis. This finding suggests a bidirectional relationship, where systemic vascular and metabolic dysregulation not only result from periodontal disease but may also act as precursors to its development. Both conditions mentioned result in a state of systemic inflammation, elevating pro-inflammatory cytokines. Dyslipidemia characterized by oxidized low-density lipoprotein and reduced high-density lipoprotein [32], amplifies this inflammation by interacting with bacterial toxins stemming from periodontal pathogens. Impaired neutrophil and macrophage function, coupled with excessive reactive oxygen species production [33], exacerbates tissue damage and delays repair. Additionally, vascular changes, including reduced microcirculation and aberrant angiogenesis, impair nutrient delivery and immune responses in the periodontium [34]. Bitencourt et al. (2023) explored the relationship between dyslipidemia and periodontitis using structural equation modeling in a large cohort of US adults participating in NHANES III [35]. Their analysis revealed that dyslipidemia had both direct and indirect effects on periodontitis, mediated by obesity, emphasizing its multifactorial nature. This aligns with our findings, which highlight the predictive strength of disorders of lipid metabolism for periodontitis.

Such a bidirectional framework underscores the interconnected nature of oral and systemic health, pointing to shared inflammatory pathways and metabolic dysregulation as central drivers. By reversing the commonly studied causal direction, these results broaden the understanding of the systemic–periodontal interplay and emphasize the need to consider systemic health conditions, such as atherosclerosis and dyslipidemia, in periodontal disease prevention and management strategies. However, systemic health does not fully account for the risk of periodontal disease as certain intraoral diseases also emerged as significant risk factors for periodontal disease in this study. Excessive attrition, abrasion, erosion, and pathological resorption of teeth are often indicative of chronic mechanical or chemical insults [36]. These conditions may reflect long-term behavioral factors such as bruxism, improper toothbrushing techniques, dietary acid exposure, or poor oral hygiene practices. Additionally, deposits on teeth and post-eruptive color changes can signal plaque accumulation and enamel degradation, serving as indirect markers of suboptimal oral care. These structural anomalies, while not directly inflammatory, can create a favorable environment for bacterial colonization and subsequent breakdown of periodontal tissue [37].

In contrast, pulpitis, periapical abscesses, and chronic apical periodontitis represent inflammatory and infectious processes affecting the dental pulp and surrounding periapical tissues. These conditions are often the result of untreated caries or trauma, leading to localized infections that may exacerbate systemic inflammatory responses. Similarly, occlusal trauma reflects mechanical stressors on teeth and supporting structures, compromising periodontal stability and contributing to progressive tissue damage, especially when compounded by bacterial and inflammatory factors. Ríos et al. (2021), similarly to the current study’s results, found that occlusal trauma is significantly associated with periodontitis even after adjusting for multiple confounders [38].

Developmental variations in tooth morphology, such as enamel hypoplasia or dentin defects, can create anatomical features that favor plaque accumulation and calcified deposits, making mechanical plaque control challenging even for diligent patients. These anomalies can hasten the onset and progression of periodontal disease by providing niches for bacterial biofilm formation and exacerbating localized gingival inflammation and periodontal attachment loss [39]. Similarly, the 2017 World Workshop on the Classification of Periodontal and Peri-Implant Diseases and Conditions highlighted that anatomical factors related to tooth morphology are associated with biofilm-induced gingival inflammation and loss of periodontal supporting tissues. These developmental or acquired conditions can predispose patients to periodontal diseases by modifying the periodontal attachment apparatus and amplifying inflammatory responses [40]. Recognizing and addressing these morphological factors early may improve the prognosis for affected teeth and mitigate their impact on periodontal health.

This study’s strengths lie in its use of real-world clinical data collected over a seven-year follow-up period, providing a pragmatic insight into the natural progression of periodontal disease. Diagnoses were based on ICD-10 codes by physicians, ensuring clinical relevance. The large sample size enhances statistical power, while laboratory-measured biomarkers offer objective assessments of systemic health. The longitudinal design enables the analysis of causal and temporal relationships, and the application of robust time-to-event models ensures methodological rigor by adjusting for both systemic and intraoral health factors.

Limitations include the use of data from a single center, which may limit the generalizability of findings to broader populations. The reliance on ICD-10 codes does not provide detailed information on the severity or grade of the diseases studied. The absence of socioeconomic and demographic variables in the dataset precludes the exploration of their potential confounding effects. An important confounding factor, smoking, was not included in the analysis as the clinical database did not contain information on smoking habits or status. Smoking is a well-established risk factor for periodontitis, and its exclusion may influence the robustness of the findings. The retrospective nature of the study introduces inherent potential biases in data availability and recording, which may impact the interpretation of the results, and not all patients had data available for biomarkers such as CRP and eGFR, which may limit the strength of associations observed for these variables. Lastly, unmeasured confounding factors and the observational nature of the study may influence the interpretation of causality.

## 4. Materials and Methods

### 4.1. Data Cleaning and Processing

This retrospective longitudinal study utilized clinical data collected from hospital records at the University of Debrecen Hospital between 2007 and 2022 to assess predictors of periodontitis. Due to inconsistencies in early data and a limited sample size for periodontitis diagnoses before 2015, records prior to 2015 were excluded. The study’s baseline year was established as 2015, and any participants diagnosed with periodontitis on or before this year were excluded to ensure a clean baseline. Participants were included if they had either a diagnosis of periodontitis, identified through ICD-10 codes, or were part of the general hospital population with recorded dental diagnoses or procedures without a prior diagnosis of periodontitis.

The resulting dataset comprised 23,742 records corresponding to 4833 unique participants. Among these, 5264 records indicated a diagnosis of periodontitis. Periodontitis was defined using a composite International Classification of Diseases, 10th Revision (ICD-10) variable [41], including codes K05, K05.2, K05.3, K05.4, K05.5, and K05.6. For participants with a periodontitis diagnosis, the year of diagnosis marked the end of follow-up. For censored participants, follow-up was defined as the last year with available data or the study endpoint in 2022. This design allowed for a robust evaluation of the temporal relationships between inflammatory biomarkers, systemic health variables, and the incidence of periodontitis in a large clinical population.

### 4.2. Variables of Interest

The variables included in this analysis encompassed demographic, clinical, biomarker, and systemic and oral health-related factors. Gender was coded as a binary variable (male or female), and age was treated as a continuous variable in years. Biomarkers included CRP, measured in mg/L and dichotomized at a cutoff of 15 mg/L (≤15 mg/L vs. >15 mg/L) [42]; eGFR, measured in mL/min/1.73 m^2^ and dichotomized at 60 mL/min/1.73 m^2^ (≥60 vs. <60) [43]; and liver function enzymes, glutamic oxaloacetic transaminase (GOT) and glutamic pyruvic transaminase (GPT), both measured in international units per liter (IU/L) and dichotomized with a cutoff of 40 IU/L (≤40 IU/L vs. >40 IU/L). Systemic and oral health conditions were included as binary variables (absent vs. present), based on the corresponding ICD-10 codes, and comprised disorders of lipoprotein metabolism (E78), atherosclerosis (I70), disorders of tooth development and eruption (K00), other diseases of the hard tissues of teeth (K03), diseases of pulp and periapical tissues (K04), other disorders of gingiva and edentulous alveolar ridge (K06), other specified disorders of teeth and supporting structures (K08), type 2 diabetes mellitus (E11), and essential hypertension (I10). This standardized approach to variable coding ensured consistency and facilitated the exploration of their associations with periodontitis.

### 4.3. Statistical Analysis

Baseline characteristics were determined based on the first recorded appearance of each participant in the dataset. Age was analyzed as a continuous variable and presented as the mean and standard deviation (SD), as well as the median and interquartile range (IQR). All other variables, being categorical, were summarized as frequencies and proportions.

#### 4.3.1. Univariate Analysis and Visualization

Univariate Cox proportional hazards regression models were applied to assess the relationship between individual covariates and the time to diagnosis of periodontitis [44]. This method was employed to evaluate the equality of survival functions across different levels of categorical variables. It provided outputs in terms of observed and expected events, relative hazards, and corresponding *p*-values, offering insights into the independent contribution of each variable to the hazard of periodontitis.

The Cox proportional hazards model for a single covariate $X_{i}$ is represented as follows:

(1) $$h\left(t|X\right)=h_{0}\left(t\right)\;\exp(\beta_{i}X_{i})$$

where $h\left(t|X\right)$ is the hazard at time t, $h_{0}\left(t\right)$ is the baseline hazard, $\beta_{i}$ represents the coefficient for covariate $X_{i}$, and $X_{i}$ is the covariate.

Kaplan–Meier survival curves were generated for significant variables [45]. These curves depicted both observed and predicted survival probabilities, stratified by the levels of the covariates, providing a graphical assessment of differences in survival distributions. The Kaplan–Meier estimator calculates survival probabilities at specific time points, defined as

(2) $$\hat{s}\left(t\right)=\underset{t_{i}\leq t}{\prod }\left(1-\frac{{\mathrm{d}}_{i}}{n_{i}}\right)$$

where,

$\hat{s}\left(t\right)$ is the estimated survival probability at time  t;
${\mathrm{d}}_{i}$ is the number of events (e.g., diagnosis of periodontitis) at time $t_{i}$;
$n_{i}$ is the number of participants at risk just before time $t_{i}.$

This stepwise estimator accounts for survival probabilities by multiplying the conditional probabilities of surviving past each time point where an event occurs.

Proportional hazards assumptions for the Cox models were evaluated using Schoenfeld residuals [46], with both global and covariate-specific diagnostics conducted to ensure model validity. Any deviations from these assumptions were noted and appropriately addressed.

Cumulative hazard analysis was performed to evaluate the aggregate risk of developing periodontitis over time among various subgroups. The cumulative hazard function, $\hat{H}\left(t\right)$, was estimated using the Nelson–Aalen estimator, which provides a non-parametric estimate of cumulative hazard [47,48,49]. The Nelson–Aalen estimator is defined as

(3) $$\hat{H}\left(t\right)=\underset{t_{i}\leq t}{\sum }\frac{{\mathrm{d}}_{i}}{n_{i}}$$

where,

$\hat{H}\left(t\right)$ is the cumulative hazard estimate and time t;
$t_{i}$ denotes the observed event times;
  ${\mathrm{d}}_{i}$ is the number of events (periodontitis diagnoses) at $t_{i}$;
$n_{i}$ is the number of individuals at risk just prior to $t_{i}$.

This method accounts for right-censoring and provides a robust approach to estimating the cumulative hazard. The cumulative hazard curves were visualized, stratified by key covariates such as gender, CRP categories, eGFR, and systemic or oral health-related conditions.

#### 4.3.2. Parametric Survival Modelling Using the Gompertz Distribution

To model the time to periodontitis diagnosis, a series of parametric survival models were considered, including the Gompertz, Weibull, log-normal, log-logistic, generalized gamma, and Cox proportional hazards models. Model selection was based on the Akaike Information Criterion (AIC) and Bayesian Information Criterion (BIC), which assess model fit while penalizing for model complexity [50,51]. Among the models tested, the Gompertz distribution exhibited the lowest AIC and BIC values, indicating superior fit to the data [52]. Visual inspection of the cumulative hazard and survival functions further supported the appropriateness of the Gompertz model by aligning closely with the observed hazard trends.

The Gompertz model is particularly suitable for survival data with monotonically increasing or decreasing hazard rates over time [53,54], aligning with the hypothesized hazard trajectory of periodontitis in this population. Unlike the Cox model, which assumes a non-parametric baseline hazard, the Gompertz model provides a fully parametric framework, offering a more interpretable hazard structure and better extrapolation capabilities.

The Gompertz distribution assumes that the hazard function, $h\left(t\right)$, increases (or decreases) exponentially over time and is defined as

(4) $$h\left(t\right)=\lambda e^{\gamma t}$$

where,

λ > 0 is the scale parameter, governing the baseline hazard;
γ is the shape of the parameter, determining the rate of hazard change over time.

The corresponding survival function and cumulative hazard function are given by

(5) $$S\left(t\right)=\exp\left(-\frac{\lambda }{\gamma }(e^{\gamma t}-1)\right)$$

(6) $$H\left(t\right)=\frac{\lambda }{\gamma }\;(e^{\gamma t}-1)$$

where $S\left(t\right)$ represents the probability of surviving beyond time t, and $H\left(t\right)$ quantifies the cumulative hazard up to time t.

Model output from the Gompertz survival analysis was presented as hazard ratios (HRs) with corresponding 95% confidence intervals (CIs). A hazard ratio greater than 1 indicates an increased risk, while a hazard ratio less than 1 suggests a protective effect. The threshold for statistical significance across all analyses was set at *p <* 0.05. All analyses and graphical visualizations were performed using Stata software version 17.0 [55]. This included the generation of survival and hazard estimates, as well as comparative assessments of model fit and graphical diagnostics.

## 5. Conclusions

Biomarkers such as CRP and eGFR could demonstrate potential predictive value in the early identification of periodontitis, facilitating timely interventions to prevent tooth loss and limit the progression of associated systemic conditions. Patients with chronic kidney disease, atherosclerotic heart disease, and disorders of lipoprotein metabolism are particularly at increased risk, highlighting the need for an integrated approach to periodontal care that considers these systemic health factors along with oral diseases. Clinicians should also prioritize the early detection and management of systemic and oral health conditions to mitigate periodontal disease progression. Further research is warranted to explore the underlying mechanisms and refine predictive models, aiming to enhance personalized care and improve outcomes in periodontal health.

## Figures and Tables

**Table 1:** Baseline characteristics of study population at start year of follow-up (2015).

Variable (ICD-10 Code)	Categories	*n* (%)
Age	Mean (SD)	50.43 (15.40)
	Median (IQR)	51 (40–62)
	Min–Max	0–89
Gender	Male	2193 (45.40%)
	Female	2637 (54.60%)
C-reactive protein	Normal (≤15)	295 (74.31%)
	High (>15)	102 (25.69%)
eGFR	Normal (eGFR ≥ 60)	317 (80.25%)
	Kidney Disease (eGFR < 60)	78 (19.75%)
GOT	Normal	528 (91.83%)
	Elevated	47 (8.17%)
GPT	Normal	576 (87.67%)
	Elevated	81 (12.33%)
Disorders of lipoprotein metabolism (E78)	Absent	4704 (97.33%)
	Present	129 (2.67%)
Atherosclerosis (I70)	Absent	4796 (99.23%)
	Present	37 (0.77%)
Disorders of tooth development and eruption (K00)	Absent	4767 (98.63%)
	Present	66 (1.37%)
Other diseases of hard tissues of teeth (K03)	Absent	4063 (84.07%)
	Present	770 (15.93%)
Diseases of pulp and periapical tissues (K04)	Absent	4686 (96.96%)
	Present	147 (3.04%)
Other disorders of gingiva and edentulous alveolar ridge (K06)	Absent	4756 (98.41%)
	Present	77 (1.59%)
Other specified disorders of teeth and supporting structures (K08)	Absent	4666 (96.54%)
	Present	167 (3.46%)
Type 2 diabetes mellitus (E11)	Absent	4743 (98.14%)
	Present	90 (1.86%)
Essential hypertension (I10)	Absent	4497 (93.05%)
	Present	336 (6.95%)

Note: All values are presented as *n*(%) unless otherwise indicated. eGFR, estimated glomerular filtration rate; GOT, glutamate oxaloacetate transaminase; GPT, glutamate pyruvate transaminase.

**Table 2:** Cox regression-based tests for equality of survivor functions.

Variable (ICD-10 Code)	Categories	Observed Events	Expected Events	Relative Hazard	*p*-Value
Gender	Male	2361	2181.13	1.09	**<0.001**
	Female	2900	3079.87	0.94	
C-reactive protein	Normal (≤15)	326	359.26	0.92	**<0.001**
	High (>15)	138	104.74	1.34	
eGFR	Normal (≥60)	284	302.41	0.94	0.072
	Kidney Disease (<60)	174	155.59	1.12	
GOT	Normal	481	490.68	0.98	0.125
	Elevated	50	40.32	1.24	
GPT	Normal	508	527.06	0.97	**0.024**
	Elevated	96	76.94	1.25	
Disorders of lipoprotein metabolism (E78)	No	5141	5164.42	1	**0.022**
	Yes	123	99.58	1.24	
Atherosclerosis (I70)	No	5205	5224.54	1	**0.004**
	Yes	59	39.46	1.5	
Disorders of tooth development and eruption (K00)	No	4916	5136.64	0.97	**<0.001**
	Yes	348	127.36	2.79	
Other diseases of hard tissues of teeth (K03)	No	3800	4881.88	0.89	**<0.001**
	Yes	1464	382.12	4.5	
Diseases of pulp and periapical tissues (K04)	No	4813	5110.94	0.97	**<0.001**
	Yes	451	153.06	3.03	
Other disorders of gingiva and edentulous alveolar ridge (K06)	No	5071	5218.26	0.99	**<0.001**
	Yes	193	45.74	4.3	
Other specified disorders of teeth and supporting structures (K08)	No	4739	5132.68	0.96	**<0.001**
	Yes	525	131.32	4.19	
Type 2 diabetes mellitus (E11)	No	5173	5198.9	1	**0.002**
	Yes	91	65.1	1.4	
Essential hypertension (I10)	No	4983	5069.43	0.99	**<0.001**
	Yes	281	194.57	1.45	

Note: The table displays the results of Cox regression-based tests for equality of survivor functions for each covariate. Relative hazard ratios compare the risk of events across groups, with *p* < 0.05 indicating statistical significance. eGFR, estimated glomerular filtration rate; GOT, glutamate oxaloacetate transaminase; GPT, glutamate pyruvate transaminase.

**Table 3:** Hazard ratios from the Gompertz model.

Variable (ICD-10 Code)	Categories (Reference)	Hazard Ratio (HR) [95% CI]	*p*-Value
Age	Continuous	**0.99 [0.98–0.99]**	**0.001**
Gender	Female (ref: Male)	0.79 [0.63–1.00]	0.054
C-reactive protein	High (>15) (ref: ≤15)	**1.36 [1.05–1.77]**	**0.02**
eGFR	<60 (ref: ≥60)	**1.39 [1.08–1.78]**	**0.01**
GOT	Elevated (ref: Normal)	0.89 [0.57–1.38]	0.604
GPT	Elevated (ref: Normal)	1.04 [0.72–1.48]	0.85
Disorders of lipoprotein metabolism (E78)	Present (ref: Absent)	0.75 [0.40–1.40]	0.361
Atherosclerosis (I70)	Present (ref: Absent)	**2.12 [1.11–4.06]**	**0.023**
Disorders of tooth development and eruption (K00)	Present (ref: Absent)	1.09 [0.67–1.78]	0.729
Other diseases of hard tissues of teeth (K03)	Present (ref: Absent)	**7.30 [5.45–9.78]**	**<0.001**
Diseases of pulp and periapical tissues (K04)	Present (ref: Absent)	**1.91 [1.14–3.20]**	**0.014**
Other disorders of gingiva and edentulous alveolar ridge (K06)	Present (ref: Absent)	1.41 [0.68–2.94]	0.357
Other specified disorders of teeth and supporting structures (K08)	Present (ref: Absent)	**3.02 [2.05–4.43]**	**<0.001**
Type 2 diabetes mellitus (E11)	Present (ref: Absent)	1.16 [0.59–2.29]	0.673
Essential hypertension (I10)	Present (ref: Absent)	1.07 [0.68–1.69]	0.755

Note: Hazard ratios (HRs) with 95% confidence intervals (CI) were estimated using a Gompertz regression model. Significant covariates (*p* < 0.05) are highlighted. Abbreviations: eGFR, estimated glomerular filtration rate; GOT, glutamate oxaloacetate transaminase; GPT, glutamate pyruvate transaminase.

## Data Availability

The data presented in this study are available on request from the corresponding author. The data are not publicly available due to institutional restrictions.
